# Use of columnar cacti in the Tehuacán Valley, Mexico: perspectives for sustainable management of non-timber forest products

**DOI:** 10.1186/1746-4269-10-79

**Published:** 2014-12-23

**Authors:** Edgar Pérez-Negrón, Patricia Dávila, Alejandro Casas

**Affiliations:** Centro de Investigaciones en Ecosistemas, UNAM, Campus Morelia. Apartado Postal 27-3, Santa María de Guido Morelia, 89190 Michoacán, México; UBIPRO, Facultad de Estudios Superiores Iztacala, UNAM, Apartado Postal 54090 (Los Reyes Iztacala), Tlalnepantla, Estado de México, México

**Keywords:** Arid zones forests, Columnar cacti, Non-timber forest products, Pitaya, Sustainable harvest

## Abstract

**Background:**

TEK, ecological and economic aspects of columnar cacti were studied in the Tehuacán Valley, Mexico to design sustainable regimes of fruit harvest. We analysed the amounts of edible fruit, seeds and flowers produced per hectare of cardonal, jiotillal and tetechera forests, their economic value and actual extraction rates, hypothesizing that the economic benefits of these NTFP would potentially be comparable to maize agriculture, which involves forest removal.

**Methods:**

Our study comprised the whole territory of the community of Quiotepec, Oaxaca. Sustainable gathering rates were analysed through population dynamics models and simulations of harvesting regimes (10%, 25%, and 50% of fruit gathered) per hectare of forest type. We used estimations on economic benefit and ecological impact of these scenarios to evaluate their relative sustainability, compared with maize agroforestry systems harbouring 2-47% of vegetation cover.

**Results:**

For the whole territory, the total annual fruit production is 509.3 ton of *Pachycereus weberi*, 267.4 ton of *Neobuxbaumia tetetzo*, 99.5 ton of *Escontria chiotilla*, and 8.1 ton of *Myrtillocactus geometrizans*. The total economic value of fruits per hectare was $315.00 U.S. dollars for cardonal, $244.60 for jiotillal, and $113.80 for tetechera, whereas rainfed agriculture of maize was on average $945.52. Demographic models for *E. chiotilla* and *N. tetetzo* indicate that 70% and 95% of fruit harvesting, respectively maintain λ > 1, but these harvest rates cannot be recommendable since the models do not consider the high inter-annual environmental variations and the non-estimated amount of fruit consumed by natural frugivorous. Extracting 25% of fruit is ecologically more sustainable, but with low economic benefits. Agroforestry systems maintaining the higher vegetation cover provide economic benefits from agriculture and forest resources.

**Conclusions:**

Combining forest extraction and agroforestry systems are ideal scenarios to sustainable fruit harvest programmes. In addition, fair commerce of transformed products would substantially favour goals of sustainable management.

## Introduction

Arid and semiarid zones cover nearly one-half of the Mexican territory, lodging a high biological and cultural diversity. Rzedowski [1] estimated that in these zones occur nearly 6,000 species of vascular plants, approximately 20% of the Mexican flora, and 60% of them being endemic. Casas *et al*. [2] identified 30 indigenous groups living in Mexican arid zones, out of the 58 registered in the whole country [3]. Human presence in arid zones of Mexico is dated nearly 12,000 years old [4], and throughout such a long history indigenous peoples have developed a rich knowledge and experience of using and managing arid ecosystems and numerous species that compose them. However, arid ecosystems have among the highest levels of complexity and fragility to anthropic perturbation, and are among those with the highest risk to disappear [5]. The long life cycles and slow growth rates characterizing species inhabiting these areas determine extraordinary tardily recovery of ecosystems after disturbance [6, 7]. Also in these zones, particularly in tropical regions, biotic communities maintain specialized interactions and, therefore, the effects of perturbation on particular species may have drastic consequences on entire ecosystems. For instance, decreasing numbers of arboreal and shrubby species may determine the depletion or local extinction of numerous succulent plant species, which are not able to survive in arid environments during the first stages of their life cycle in absence of nurse plants [8]. Other interactions such as pollination and seed dispersal may be specialized and the scarcity or absence of pollinators and seed dispersers on one hand, or plant resources for maintaining these organisms on the other hand, may cause drastic negative effects on the biotic communities of arid ecosystems [9]. For this reason, it is particularly relevant to design careful strategies of sustainable use of biotic resources and ecosystems of these regions.

The Tehuacán-Cuicatlán Valley is an important arid region of México. Its territory is approximately 10,000 km^2^, but it lodges a high biological diversity represented by more than 3,000 vascular plant species [10–12]. The region has also a high cultural diversity, represented by eight indigenous ethnic groups (Nahua, Popoloca, Mazatec, Chinantec, Mixtec, Ixcatec, Chocho and Cuicatec). Ethnobotanical studies have revealed that local peoples know and use more than 1,600 plant species [11], about 90% of them occurring in the wild as part of 36 different types of forests. Gathering of forest products continues being crucial for subsistence of rural households; for instance, nearly 12% of annual diet of people, and nearly 90% of requirements of fodder and fuel for cooking is supplied by non-crop plant species [13]. Nearly 600 species have been documented to receive some management form other than simple gathering [10, 13, 14] either by cultivation or silviculture in agroforestry systems, but the remaining species are extracted from the forests, some of them with negative impacting practices. Therefore, developing management techniques for sustainable harvesting of forest products is a priority for regional programmes of both conservation of natural ecosystems and human well-being.

Columnar cacti are particularly important plant resources in the region; 20 species of them are dominant components of different vegetation types described as columnar cacti forests [15], which occupy the most extended area of the Tehuacán Valley. All species of columnar cacti are useful for their edible fruits, and some of them provide edible flower buds and seeds, as well as fodder, fuelwood, wood for construction, and live fences [2]. There is archaeological evidence that humans utilized these plants since the first stages of occupation of the region [4], and some of them still have economic importance at the present [16].

The higher impact on columnar cactus forests of the region is determined by clearing of vegetation to establish agricultural fields [17], as well as free raising of cattle and goats, which forage nurse plants and trample seedlings and sapling plants in forests [18]. However, agriculture and livestock are the basic economical support of local rural households. Conservative production activities, such as extraction and commercialization of non-timber forest products (NTFP) may be alternative activities favouring concealing conservation and human well-being. Nevertheless, to be viable these activities should be economically profitable [19]. When NTFP are under sustainable harvest, the activity may have a low impact on natural ecosystems, compared with practices such as agriculture, livestock or intensive wood extraction. In addition, the economic value of forest resources may be an important incentive for its conservation [20]. In this study, we analyze the viability of alternative activities associated to the extraction of useful products of columnar cacti, which are among the NTFP with higher economic value in the region [13, 16].

Ethnobotanical studies have documented that fruits of most columnar cacti species are used to complement the diet of rural people in different seasons throughout the year [2, 21]. In addition, fruits of some species are under active exchange in traditional markets [16]. For instance, [22] documented that in homegardens *Stenocereus stellatus* may produce 3.30 ton of fruit per ha, and such production along with that from wild populations allows covering the households consumption requirements and surpluses for commercialization. Mercado and Granados [23] identified the Tehuacán Valley and La Mixteca, as the regions of Mexico most producing and commercializing columnar cacti fruit, mainly from the species *Stenocereus pruinosus*, *S. stellatus*, *Escontria chiotilla*, *Polaskia chichipe*, and *Mirtyllocactus geometrizans*.

In a previous study in the Tehuacán Valley, our research group [24] identified that fruits of *E. chiotilla*, *P. weberi*, *N. tetetzo*, and *M. geometrizans*, are important edible resources in the village of Quiotepec, Oaxaca, which also allow monetary incomes to local people. Fruits of these species are mainly commercialized fresh in the regional markets, but some households practice different forms of processing part of them. For instance, [25] documented that people of the neighboring village of Coxcatlán make use of *E. chiotilla* fruits to prepare ice cream and jellies traded at local level. Also in the Tehuacán Valley, [26] found that fruits of *P. chichipe* are dried to produce “pasado” (raisin) fruit. Casas *et al*. [2] documented that flower buds of *N. tetetzo* are commercialized cooked and prepared with vinegar as conserve. In addition, fruits of *M. geometrizans* and *M. schenckii* are consumed fresh, prepared as ice cream, dried as raisins or mixed with aguardiente to prepare spirits.

In the village of Quiotepec, agriculture is the main economic activity and about 80% of the currently active agricultural plots are irrigated systems from the Cacahuatal, Salado, and Grande rivers. Most irrigated agriculture produces commercial fruit (lemon, mango, sapodilla, and avocado), and part of the maize consumed by the local households. However, nearly 190 hectares of transformed forests (about 40% of both active and fallow agricultural area) are rainfed agroforestry systems producing maize [24]. Forest areas are communal property and clearing land for agriculture requires permits from local authorities, which frequently causes conflicts among ejidatarios and comuneros farmers. Ejidal and communal are collective forms of land tenure in Mexico but they have different rights on communal and ejidal land and forest resources and, consequently, they continually negotiate rules of access to land since the 1960’s [27]. These sectors have tensions especially in relation to clearing areas for agriculture. After the decree of the Tehuacán-Cuicatlán biosphere reserve in 1998, the authorities established regulations and production programs to stop forest clearing in order to favor biodiversity conservation. However, this policy sometimes has increased the tension between ejidatarios and comuneros. Constructing production systems making compatible both conservation and economic benefits is an important issue for all actors as a way to diminish conflicts. Such actions could be helpful in Quiotepec, as well as in other rural villages of the Tehuacán Valley. This challenge, nevertheless, requires a process of constructing alternative management systems, building agreements among people, innovating techniques, and monitoring the actions to improve them continually. Participation of local people and their institutions, considering their views, knowledge, technical experience and their abilities for agreements is crucial. Also crucial is the participation of the academic sector (mainly researchers conducting ecological, economic, ethnobiological and institutions studies), and conservation authorities who must respect local agreements and support conservation and sustainable production programs. In this study, we summarize ecological and economic information intended to contribute in such a process.

We hypothesized that the high densities of columnar cacti that are dominant species in some forests (e.g. *P. weberi* in cardonal, *E. chiotilla* in jiotillal, and *N. tetetzo* in tetechera forests) would allow sustainable fruit extraction, whose economic benefits may be similar or even higher than from rainfed maize agriculture, which requires deforestation. If this hypothesis is correct, fruit gathering could become a viable option to contribute to decrease the deforestation associated to agricultural practices and to obtain economic benefits with low ecological impact. Therefore, our study aimed to: (1) Document annual production and the economic value of fruits produced in columnar cacti forests. (2) Identify sustainable extraction rates according to ecological criteria. (3) Integrate ecological and economic criteria to evaluate sustainability of fruit gathering regimes, comparing them with the economic value of rainfed production systems of maize.

## Materials and methods

### Study area

**Figure 1:**
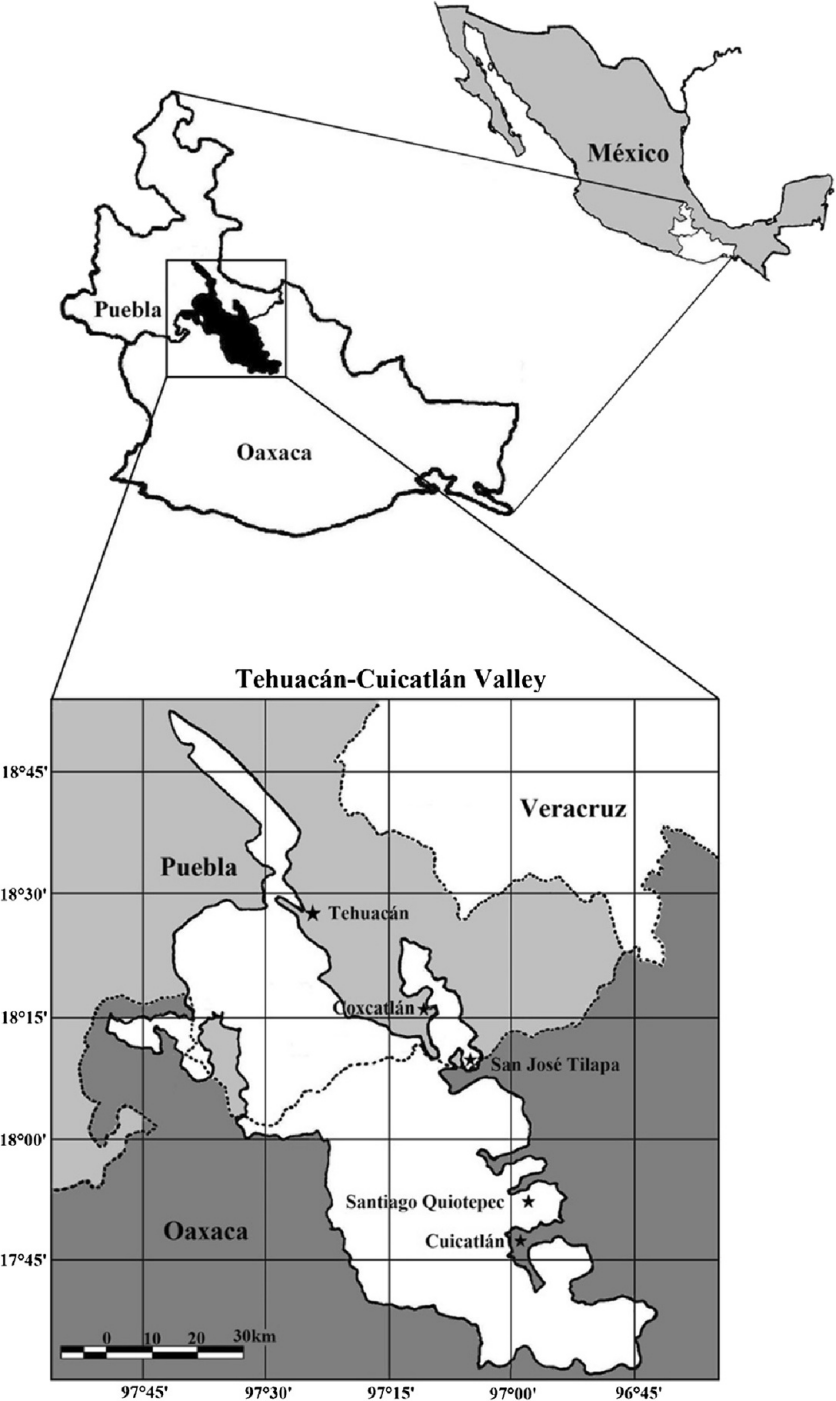
**Geographic location of the territory of Quiotepec, in the municipality of Cuicatlán, Oaxaca, within the biosphere reserve Tehuacán-Cuicatlán, central Mexico.**

The village of Quiotepec is located at the northeast of the state of Oaxaca, in the municipality of Cuicatlán, within the Tehuacán-Cuicatlán biosphere reserve (Figure 1). In total, 84 households inhabit the village, which practice agriculture, livestock, and extraction of forest products for sustaining their lives. The territory of Quiotepec has elevations from 545 to 1,400 m, with semiarid climate averaging 508.4 mm of annual precipitation and annual mean temperature of 25.1°C [28]. The total area of the territory is 49.3 km^2^, covered by a mosaic of vegetation types, mainly tropical dry and columnar cacti forests, as described by [15]. One of the cacti forests is jiotillal, dominated by *Escontria chiotilla*, another is tetechera dominated by *Neobuxbaumia tetetzo*, and the other cardonal dominated by *Pachycereus weberi*. In addition, cuajiotal dominated by *Bursera* spp., and riparian vegetation dominated by *Astianthus biminalis* and *Taxodium mucronatum* are important vegetation types [24].

### Species and forest types studied

The species studied are candelabriform columnar cacti that are dominant elements in cardonal, jiotillal, and tetechera forests. *P. weberi* is the most robust cactus of central Mexico, it is pollinated by bats and produces fruit from February to March [29]; *N. tetetzo* is also bat pollinated and produces flowers in April and fruits from May to June [30]. Bees pollinate *E. chiotilla*, which produces fruits throughout the year although the higher production occurs between May and August [31]; bees also pollinate *M. geometrizans* flowers whose mature fruits are available from April to June. The four species studied occur in cardonal and tetechera forests, but *N. tetetzo* is absent in jiotillal.

### Ethnobotanical interviews and economic importance

Before the research was conducted, we asked permits from the authorities of the Biosphere Reserve, from Communal and Ejidal authorities, and explained in the corresponding Communal and Ejidal meetings the purpose of our study to all men and women of Quiotepec. A survey was conducted to family heads of 25 randomly selected households (nearly 30% of all households in the village), in order to evaluate the consumption of fruit, flower buds and seeds of columnar cacti as part of the people’s diet, as well as commercialization of these useful products and the role of this activity in the households economy. For randomizing the households sampled, we consulted the authorities’ archives. Every household appeared with a number in a checklist. Through a calculator, we generated random numbers for choosing the household to be interviewed. When a household was absent or had no disposition to collaborate with the study, we generated a new random number.

Although all households interviewed in this study said that they occasionally sell fruits or seeds of cacti, a previous study (Pérez-Negrón and Casas 2007) allowed estimating that nearly 75% of households trade cacti products either occasionally or commonly. We asked to local people of Quiotepec questions regarding amounts of fruits, flower buds or seeds extracted per species per production season, the proportion of products directly consumed, that commercialized, the destination of the commercialized products, their prices, among other aspects. We also included ethnobotanical questions in relation to resources’ preference for local consumption and commercialization, perception of impact of gathering on ecosystems where the collected plant products are extracted, the risk of plant populations used, management practices to decrease such risk, among other issues.

We converted prices from Mexican pesos into U.S. dollars, averaging 13 pesos per dollar, according to the exchange rate during the study period. We also conducted interviews to a total of 50 fruit traders in the main regional markets [those of the villages of Cuicatlán (6 traders), San Juan de los Cúes (4 traders), Tecomavaca (4 traders), Teotitlán (8 traders), Zinacatepec 9 traders), Ajalpan (10 traders), and Tehuacán (9 traders)]. These interviews evaluated the regional demand of fruit of the species studied, variation in prices throughout their production season, the provenance of the fruit, among other topics. Measure units used by traders (liters, “medida”, and “ciento” or “hundred”) were standardized by estimating their equivalence into kg.

### Fruit production and plant size structure in populations

In each forest type, we established one ha quadrants (100 × 100 m), where we monitored and evaluated the production of the main useful products of all individual plants of the four species studied throughout one year. We directly counted all fruits produced per individual plant in the sampled areas, collecting and weighing 30 mature fruits of each species, in order to estimate an average weight per fruit, as well as the average number and weight of seeds per fruit. Because of the asynchronical production of fruit, we had to follow monthly the production of flower buds, flowers in anthesis, immature fruits and mature fruits. We labelled and monitored samples of 100 flower buds, 100 flowers in anthesis and 100 immature fruits per species per site in order to estimate the proportion of each phenological state structure passing to the following one, and finally becoming a mature fruit.

By using digital orthophotos (scale 1:20,000 [32]), a topographic map (scale 1:50,000 [33]), and field trips georeferencing observation points throughout the territory of Quiotepec, we constructed a vegetation map. Ortophotos were ensambled with the program ILWIS 3.1, in order to generate a mosaic of vegetation patches and a map of polygons through which the spatial distribution and area of each vegetation type and other transformed environmental units of Quiotepec were determined. Through field trips, we corroborated the position and the status of the environmental units determined in a sample of 53 points. We estimated the total area of the vegetation types analyzed and multiplied it by the amounts of fruits and other useful products estimated per hectare in each vegetation type, in order to calculate the total production of useful products of the species studied in the whole territory of Quiotepec.

### Projection matrices of population growth

We calculated the size and biomass of all individual plants of *E. chiotilla* in the jiotillal forest plot and all individuals of *N. tetetzo* in the tetechera forest plot, in order to identify size categories of their populations. We used these data to analyze population dynamics based on demographic models available in the literature for these species studied in the region, and constructed a transition matrix for *E. chiotilla*, based on the demographic study by [34] for this species in the neighboring village of Coxcatlán (Table 1). The transition matrix for *N. tetetzo* based on the demographic study conducted by [35] in the village of Zapotitlán, within the region (Table 2). We used these transition matrices and the actual size category numbers (Table 3) as the population vector [36], in order to perform projections for estimating the finite population growth rate (λ) through the program Pop Tools 2.7 for Microsoft Excel. For perturbation analyses, we modified the fecundity rates in the transtion matrices, in order to simulate different fruit harvesting regimes and their consequences on λ.

**Table 1:** **Population transition matrix for** ***Escontria chiotilla*** **in jiotillal forest from Coxcatlán, Puebla (from** [34]**)**

	Stage 0	Stage 1	Stage 2	Stage 3	Stage 4	Stage 5	Stage 6	Stage 7	Stage 8	Stage 9	Stage 10	Stage 11	Stage 12
Stage 0	0											7.92	25.31
Stage 1	0.05	0.34											
Stage 2		0.41	0.45										
Stage 3			0.39	0.63									
Stage 4				0.33	0.55								
Stage 5					0.40	0.83							
Stage 6						0.17	0.80						
Stage 7							0.20	0.91					
Stage 8								0.09	0.65				
Stage 9									0.35	0.86			
Stage 10										0.14	0.91		
Stage 11											0.09	0.84	
Stage 12												0.16	0.88

Size categories: stage 1 = 0–0.05 m; stage 2 = 0.051- 0.100 m; stage 3 = 0.101-0.150 m; stage 4 = 0.151-0.200 m; stage 5 = 0.201-0.300 m; stage 6 = 0.301-0.500 m; stage 7 = 0.501-0.800 m; stage 8 = 0.801-1.000 m; stage 9 = 1.001-2.000 m; stage 10 = 2.001-3.000 m; stage 11 = 3.001-5.000 m; stage 12 = >5.001.

**Table 2:** **Population transition matrix for** ***Neobuxbaumia tetetzo*** **in tetechera forest from Zapotitlán, Puebla (from** [35]**])**

	Stage 1	Stage 2	Stage 3	Stage 4	Stage 5	Stage 6	Stage 7	Stage 8	Stage 9	Stage 10
Stage 1	0.76					8.7	14.9	22.4	28.2	29.3
Stage 2	0.07	0.89								
Stage 3		0.09	0.92							
Stage 4			0.07	0.96						
Stage 5				0.04	0.94					
Stage 6					0.06	0.93				
Stage 7						0.06	0.93			
Stage 8							0.06	0.95		
Stage 9								0.05	0.82	
Stage 10									0.11	0.95

Size categories: stage 1 = 0–0.150 m; stage 2 = 0.151- 0.450 m; stage 3 = 0.451-1.000 m; stage 4 = 1.001-1.500 m; stage 5 = 1.501-2.500 m; stage 6 = 2.501-3.500 m; stage 7 = 3.501- 4.500 m; stage 8 = 4.501-5.500 m; stage 9 = 5.501-6.500 m; stage 10 ≥ 6.501 m.

**Table 3:** **Size structure of** ***Escontria chiotilla*** **and** ***Neobuxbaumia tetetzo*** **in 1 ha of jiotillal and tetechera forests of Quiotepec, Oaxaca, respectively**

Stage	Length (m)	No. plants *E. chiotilla*	Length (m)	No. Plants *N. tetetzo*
1	0 - 0.05	1,700	0 – 0.150	10,160
2	0.051 - 0.100	2	0.151 – 0.450	45
3	0.101 - 0.150	3	0.451 – 1.000	30
4	0.151 - 0.200	4	1.001 – 1.500	21
5	0.201 - 0.300	4	1.501 – 2.500	67
6	0.301 - 0.500	12	2.501 – 3.500	126
7	0.501 - 0.800	13	3.501 – 4.500	227
8	0.801 - 1.000	6	4.501 – 5.500	221
9	1.001 - 2.000	38	5.501 – 6500	189
10	2.001 - 3.000	27	>6.500	76
11	3.001 - 5.000	25		
12	>5.001	20		

### Sustainability evaluations

The “Framework for Sustainability Evaluation of Natural Resources Management Systems (MESMIS for its achronym in Spanish)” developed by Masera *et al.* [37], was used to evaluate the sustainability of the different harvest rate regimes. Households directly consume the maize they produce, and commercialize surpluses within the village; stubble is widely commercialized for feeding livestock and is the main product of the agricultural system. For estimating the economic value of agricultural products, we recorded their prices in the regional market. Maize production in rainfed systems is 569 kg/ha [24], and its average economic value is $1,022.92 ($113.82 supplied by grain and $909.10 by nearly 1.8 ton of stubble) per hectare. However, households invest $77.40 in production, mainly in buying chemical products, paying extra-household labour, and renting animals for ploughing the land; we did not evaluate the value of household labour invested in the production system. Therefore, the neat value of the rainfed agricultural system is $945.52 U.S. dollars per hectare.

People of Quiotepec practice rainfed agriculture in areas with pronounced slopes, cleared for using it 2 to 4 years, with fallow periods between 2 and 10 years. Practices are conducted by households members using animal traction for tillage, seeds of local land races and, generally, chemical fertilizers and pesticides. Agroforestry systems enabling standing patches or strips of vegetation are common in the region [38–40], especially those promoting the presence of different columnar cacti species [41]. These systems maintain 2 to 47% of their area with original vegetation and lodge, on average, nearly 60% of native plant species occurring in local forests [38]. We therefore considered agroforestry systems as alternative sustainable management, evaluating the potential amount of fruit and its economic value, at different scenarios of vegetation cover maintained per hectare.

We simulated hypothetical scenarios of harvesting 10%, 25%, and 50% of cacti fruits in forests, estimating the economic value of these systems and comparing them with those of rainfed agriculture. Based on information previously analyzed [24], critical points and indicators for ecological, economic, and social aspects were established and calculated for each scenario (Table 4). With both qualitative and quantitative data, we constructed amoeba-type plots, following MESMIS [37].

**Table 4:** **Diagnosis criteria and indicators to evaluate sustainability in the systems of natural resources management analyzed**

System attribute	Critical points	Diagnosis criteria	Indicators
Productivity	Low productivity	Efficiency	Yield
	Low rentability		Cost/benefit relation
			Demand of labour hand
Equity	Low differentiated access level	Equity in access to the systems	Differential access to resources
Stability	Biological diversity	Diversity	Species richness
			Shannon diversity index
	Risk	Inter-annual rainfall variation	Variation in maize production
	System stability	Maintenance of biotic interactions	Impact on biotic interactions
Adaptability	Adoption of new systems	Capacity of technical innovation	Acceptance of the system
Autonomy	Dependence from outside	Self-sufficiency	Degree of inputs dependence

Based on the “framework for sustainability evaluation of natural resources management systems”, MESMIS [37]. Yield of the scenarios analyzed corresponds to the amount of fruits per hectare according to the percentage of fruit of each species collected in each vegetation type. The relation cost/benefit was calculated in terms of the amount of fruit and monetary incomes generated in each scenario. Labour hand demanded was calculated in terms of hours per person based on real actual observations of time invested in gathering. Differential access to resources was estimated in terms of rights and permits differently asked from local authorities to ejidatarios and comuneros. Information on biological diversity based on vegetation sampling in both forests and maize fields. Information on variation in maize production based on interviews to local people. Impact on biotic interactions is a qualitative estimation of the impact of harvesting fruits on frugivorous and granivorous species of the biotic community.

## Results

### Production of fruit and other useful products of the columnar cacti studied

Table 5 shows the amounts of useful products (fruits, flower buds and seeds) produced per species and vegetation type per hectare and in the whole territory of Quiotepec. The highest production of fruits are provided by tetechera and cardonal forests (263.20 kg/ha and 260.20 kg/ha, respectively), where the four species studied occur. In tetechera *N. tetetzo* is the dominant species and determines the highest contribution to fruit production, whereas in cardonal *P. weberi* is the dominant species and the highest provider of fruit. In jiotillal, *N. tetetzo* was absent and the highest fruit provider was the dominant species *E. chiotilla*.

**Figure 2:**
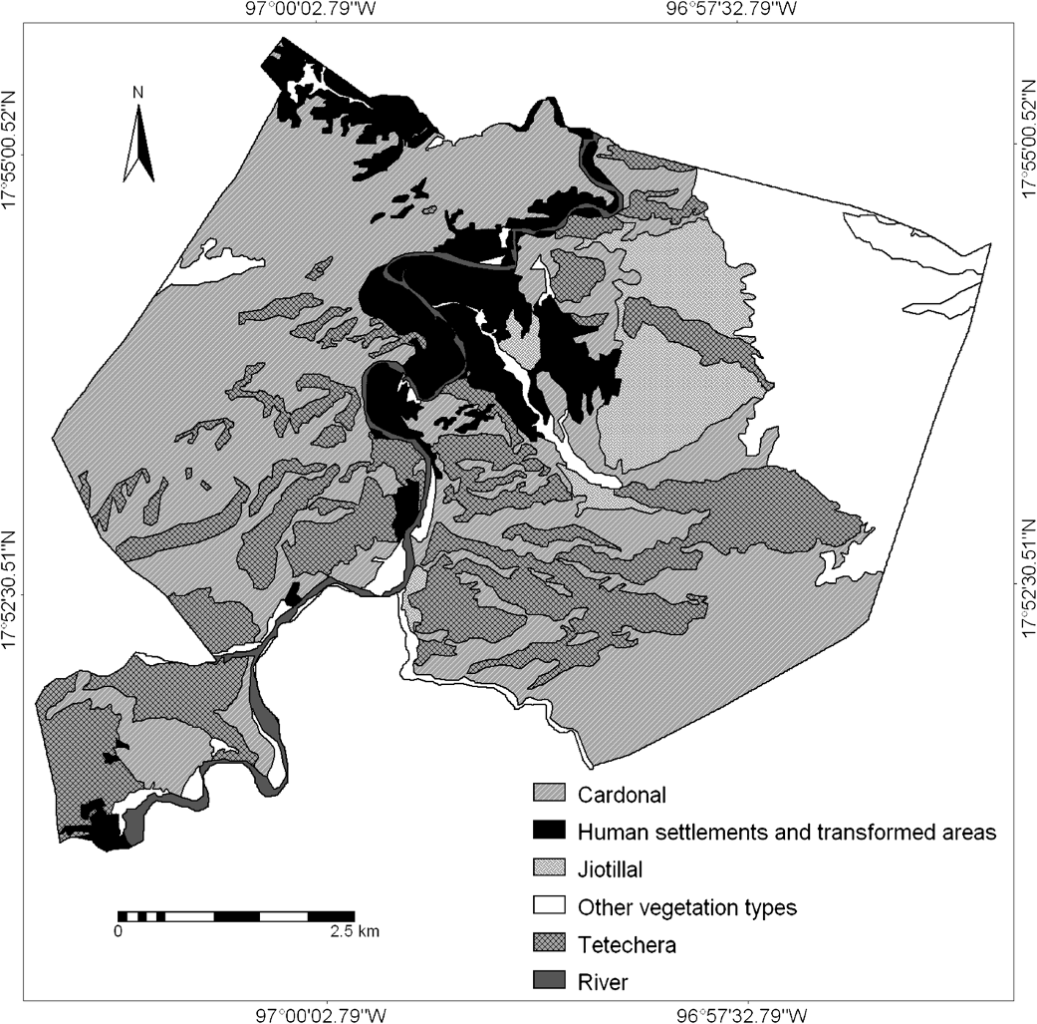
**Vegetation cover of the Quiotepec territory.** The total area of the territory of Quiotepec is 4930 hectares; the forest types studied cover the following areas: cardonal 2170 ha (44.02%), jiotillal 320 ha (6.5%), tetechera 1070 ha (21.70%).

Figure 2 indicates that cardonal is the most extended vegetation type in Quiotepec (2,170 ha), although some areas (170 ha) have been severely disturbed due to agricultural practices. It is followed by tetechera (1070 ha), which is relatively well preserved since agriculture is more difficult in areas occupied by this vegetation type. Finally, the jiotillal covers the smaller area (320 ha), and nearly 20 ha are disturbed by agricultural practices. The three environmental units studied constitute nearly 72.21% of the territory of Quiotepec. The riparian vegetation is highly transformed, since it has been the main area of human settlements and irrigated agriculture for hundreds of years.

**Table 5:** **Density of individual plants, percentage of reproductive plants, and fruit production of the columnar cacti species studied per hectare and in the whole area occupied by the different forest types analyzed**

Forest type	Parameter	*Pachycereus weberi*	*Myrtillocactus geometrizans*	*Escontria chiotilla*	*Neobuxbaumia tetetzo*
Cardonal	Density/ha	752	43	95	97
	Reproductive	18.50%	30.2%	30.5%	23.7%
	No. fruits/ha	3,647	5,705	2,521	590
	kg fruit/ha	221.60, (14.80 seeds)	2.6	29.9	6.1, (3.56 kg flower buds)
	Total ton fruit	480.90	5.60	64.80	13.20
Jiotillal	Density/ha	17	6	1855	0
	Reproductive	23.5%	66.7%	2.1%	0
	No. fruits/ha	287	9,803	8,223	0
	kg fruit/ha	17.4, (1.16 kg seeds)	4.4	97.5	0
	Total ton fruit	5.60	1.40	31.20	0
Tetechera	Density/ha	28	350	125	28,030
	Reproductive	35.7%	14.3%	6.4%	4.9%
	No. fruits/ha	351	2,288	279	23,010
	kg fruit/ha	21.3, (1.43 kg seeds)	1.0	3.3	237.6, (139.50 kg flower buds)
	Total ton fruit	22.80	1.10	3.50	254.20
Average weight per fruit (g)	60.76 ± 19.08	0.45 ± 0.18	11.85 ± 2.45	10.32 ± 2.39

According to the total area occupied by the vegetation types studied, the total production of fruit of the columnar cacti species analyzed (Table 5) is 509.3 ton of *P. weberi,* 267.4 ton of *N. tetetzo,* 99.6 ton of *E. chiotilla,* and 8.1 ton of *M. geometrizans*.

### Economic importance of columnar cacti products

Information obtained in the regional markets allowed recording fruits and seeds prices of the species studied varying during the production season, depending on their relative availability (Table 6). We did not record fruits of *M. geometrizans* in markets.

**Table 6:** **Average price (in U. S. dollars/kg) of fruits, flower buds, and seeds of** ***Escontria chiotilla Neobuxbaumia tetetzo*** **and** ***Pachycereus weberi,*** **respectively, in different markets of the region**

Market	*Escontria chiotilla*	*Neobuxbaumia tetetzo*	*Pachycereus weberi*
Ajalpan	2.75 ± 0.37 (n = 7)	0.91 ± 0.18 (n = 2)	Not found
Teotitlán	2.73 ± 0.91 (n = 2)	0.68 ± 0.23 (n = 2)	16.36 (n = 1)
Tecomavaca	1.82 ± 0.05 (n = 4)	Not found	Not found
San Juan los Cues	2.55 ± 0.05 (n = 2)	1.59 ± 0.23 (n = 2)	Not found
Coxcatlán	2.73 ± 0.53 (n = 4)	0.91 (n = 1)	Not found
Cuicatlán	1.82 ± 0.05 (n = 2)	Not found	Not found
Quiotepec	1.82 (n = 1)	Not found	Not found
Promedio general	2.32 ± 0.18 (n = 22)	1.02 ± 0.20 (n = 7)	16.36 (n = 1)

In parentheses are indicated the number of traders interviewed per market.

Currently, products of the columnar cacti gathered in Quiotepec have a value of $29.42 per household per year (Table 7), which is approximately 3.11% of the average income value obtained per household from maize produced in rainfed systems. In general, products gathered by local people are destined to direct consumption at home, but all households interviewed occasionally commercialize fruits in Quiotepec or in the city of Cuicatlán to obtain a monetary income.

**Table 7:** **Amounts and economic value of fruits of columnar cacti currently extracted in the village of Quiotepec (n = 25)**

Species	Average consumption (kg/year/household)	Income (U.S. dollars)	Average consumption (kg/year/whole village)
*Escontria chiotilla* (fruit)	9.29 (±9.62)	$21.50	761.78
*Neobuxbaumia tetetzo* (flower bud)	2.81 (±3.75)	$2.87	230.42
*Pachycereus weberi* (seeds)	0.308 (±9.04)	$5.04	25.25
*Myrtillocactus geometrizans* (fruit)	0.11 (±0.44)	-	9.02

Current extraction rates of fruits of *E. chiotilla* by all households of Quiotepec are equivalent to all fruits available in an area of 7.8 ha of jiotillal forest, which represents 2.4% of the 320 ha existing in the whole territory. Extraction rates of *N. tetetzo* products are equivalent to 1.7 ha of tetechera forest, which is 0.16% of the total 1,070 ha available, and the extraction of *P. weberi* fruits is approximately 0.08% of the 2,170 ha of cardonal forest available.

Table 8 shows that the economic value of all columnar cacti fruits available in one hectare of cardonal forest is $315.07 U.S. dollars, which represents 33.32% of the economic value of maize produced in one hectare of rainfed agriculture. Similarly, the value of all fruits per hectare of jiotillal is $244.60 (25.87% of one hectare of maize produced in rainfed systems) and tetechera $113.80 (12.08%).

**Table 8:** **Economic value of** ***Pachycereus weberi*** **seeds,** ***Escontria chiotilla*** **fruits and** ***Neobuxbaumia tetetzo*** **flower buds in cardonal, jiotillal and tetechera forests (data per hectare and in the whole surface of each forest type in the territory of Quiotepec)**

Forest type	Species	Dollars/ha	Dollars/whole territory
Cardonal	*P. weberi*	$242.18	$525,534.55
	*E. chiotilla*	$69.21	$150,173.86
	*N. tetetzo*	$3.68	$7,989.55
	*M. geometrizans*	-	-
	Total	**$315.07**	**$683,697.96**
Jiotillal	*E. chiotilla*	$225.67	$72,213.82
	*P. weberi*	$18.98	$6,074.18
	*M. geometrizans*	-	-
	Total	**$244.65**	**$78,288.00**
Tetechera	*N. tetetzo*	$142.67	$152,656.90
	*P. weberi*	$23.40	$25,038.00
	*E. chiotilla*	$7.64	$8,171.88
	*M. geometrizans*	-	-
	Total	**$173.71**	**$185,866.78**
Total			**$947,852.74**

### Gathering rates and populations maintenance

**Figure 3:**
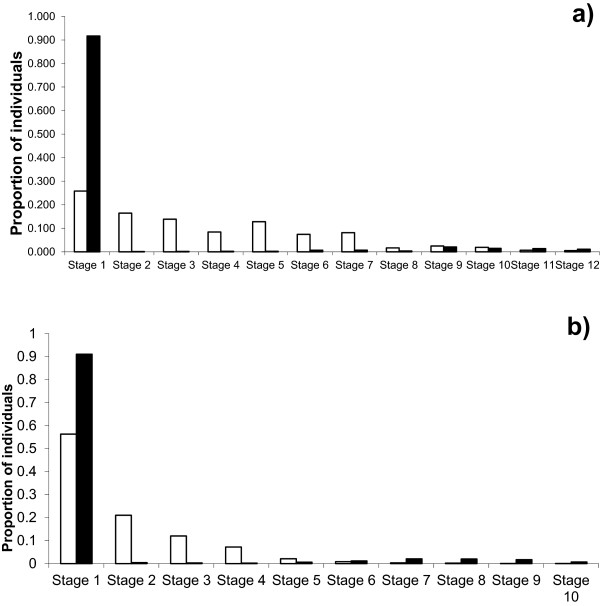
**Size structures observed (black bars) and expected according to the stable structures predicted by demographic models (white bars) for a)** ***Escontria chiotilla*** **in jiotillal forest, and b) for** ***Neobuxbaumia tetetzo*** **in tetechera forest.**

The estimated values of λ indicate that populations of *E. chiotilla* and *N. tetetzo* analyzed are growing. However, the proportion of individual sizes observed in the field differed significantly with the proportions expected in the stable size structures predicted by the models (X^2^ = 1622.206, d.f. = 11, p < 0.001 for *E. chiotilla*; X^2^ = 351974.1, d.f. = 9, p < 0.001 for *N. tetetzo*). In both cases it was notorious the high recruitment of seedlings and similar proportions of individual plants of adult stages as expected; in contrast, it is notorious the scarcity of sapling and young plants recorded in the plots sampled (Figure 3). These conditions could reflect the occurrence of a recent episodic high recruitment of seedlings on one hand, and the high mortality of young plants, probably associated to natural herbivory or pests, or because of the effect of over-grazing and or trampling by goats and cattle, on the other hand.

Perturbation analyses modifying the fecundity value (Tables 9 and 10) indicate that extraction rates up to 70% fruits in *E. chiotilla* and up to 95% fruits in *N. tetetzo* would allow maintaining λ values higher than 1. However, considering the limitations of the models used, as discussed below, as well as the undetermined amounts of fruit consumed by the community of natural frugivorous interacting with the columnar cacti species studied, our sustainability analyses considered significantly more conservative scenarios.

**Table 9:** **Disturbance analysis of** ***Escontria chiotilla*** **populations in jiotillal forest under different of fruit gathering rates**

Proportion of fruits gathered	Fecundity Reproductive 1 stage	Fecundity Reproductive 2 stage	λ
0%	7.92	25.31	1.025
10%	7.13	22.78	1.023
20%	6.34	20.25	1.021
30%	5.54	17.72	1.019
40%	4.75	15.19	1.016
50%	3.96	12.66	1.013
60%	3.17	10.12	1.009
**70%**	2.38	7.59	**1.005**
80%	1.58	5.06	0.999
90%	0.79	2.53	0.988
100%	0.000	0.000	0.910

Columns indicate the fecundity rates in reproductive individual plants determined by different gathering regimes and their effect in λ values.

**Table 10:** **Disturbance analysis of** ***Neobuxbaumia tetetzo*** **populations in tetechera forest under different fruit gathering rates**

Proportion of fruits gathered	r2	r3	Fecundity r4	r5	r6	λ
0%	8.70	14.90	22.40	28.20	29.30	1.077
10%	7.83	13.41	20.16	25.38	26.37	1.075
20%	6.96	11.92	17.92	22.56	23.44	1.073
30%	6.09	10.43	15.68	19.74	20.51	1.070
40%	5.22	8.94	13.44	16.92	17.58	1.066
50%	4.35	7.45	11.20	14.10	14.65	1.063
60%	3.48	5.96	8.96	11.28	11.72	1.058
70%	2.61	4.47	6.72	8.46	8.79	1.053
80%	1.74	2.98	4.48	5.64	5.86	1.046
90%	0.87	1.49	2.24	2.82	2.93	1.035
**95%**	0.44	0.75	1.12	1.41	1.47	**1.025**
100%	0	0	0	0	0	0.960

Columns indicate the fecundity rates in reproductive individual plants (r2 to r6) determined by different gathering regimes and their effect in λ values.

**Figure 4:**
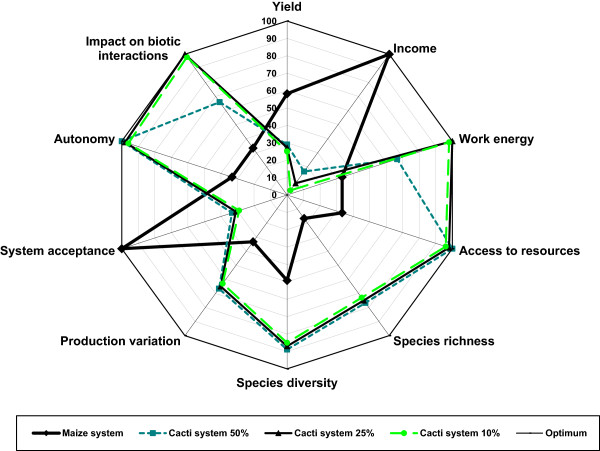
**Analysis of sustainability of productive systems in the cardonal forest using the “framework for sustainability evaluation of natural resources management systems” (MESMIS)** [37]. The sustainability degree is the sum of values of all indicators analyzed. Values close to 100% are the optimum. The higher degree of sustainability is in the non-cleared forest under gathering rates of 25% fruits of columnar cacti, followed by the system based on the extraction of 10% fruit. Maize production in rainfed system had the lowest sustainability degree.

**Figure 5:**
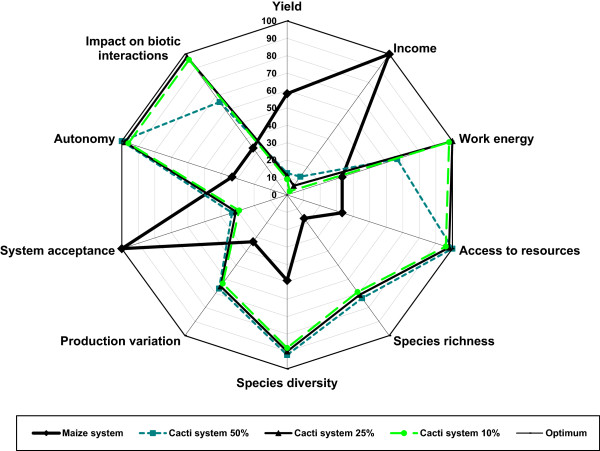
**Analysis of sustainability of productive systems in the jiotillal forest using the MESMIS** [37]. The sustainability degree is the sum of values of all indicators analyzed. The higher degree of sustainability is in the non-cleared forest under gathering rates of 25% fruits of columnar cacti, followed by the system based on the extraction of 10% fruit. Maize production in rainfed system had the lowest sustainability degree. Economic benefits from systems based on 25% and 10% of fruit extraction are lower than maize production system.

**Figure 6:**
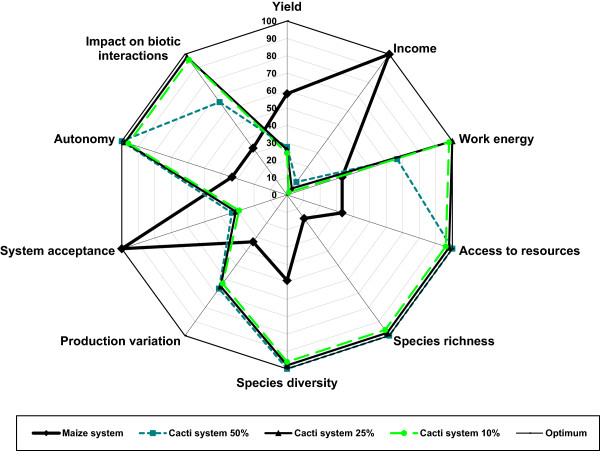
**Analysis of sustainability of productive systems in the tetechera forest using the MESMIS** [37]. Both profitability and economic benefit in production systems based on gathering of 50%, 25%, and 10% fruit of columnar cacti are lower than rainfed agricultural system of maize, but sustainability degrees of gathering systems are generally higher than agricultural system.

Figures 4, 5, and 6 show comparatively for cardonal, jiotillal and tetechera forests, respectively the following systems: (1) rainfed maize agriculture, (2) forest system extracting 50% of fruits of the species studied, (3) forest system extracting 25% of fruits, (4) forest system extracting 10% of fruits, and the (5) optimum reference system. These figures indicate that considering all ecological, economic, and social indicators used in the analysis, the harvest regime extracting 25% of fruits have the relatively higher sustainability levels in all forest types (visualized as the area of the polygon determined by the values of the variables analyzed). The scenarios of the systems harvesting 10% of fruits are effective in maintaining high levels of ecological and social indicators, whereas the relatively least sustainable system is rainfed agriculture, which has higher economic benefit but also higher negative ecological impact. Table 11 illustrates that the economic value of the agroforestry systems products is lower than that estimated for the agricultural system without any vegetation cover, but their sustainability would be expected to be higher, as long as more vegetation cover is maintained inside plots.

**Table 11:** **Economic value (U.S. dollars) of agroforestry systems with different percentages of vegetation cover, calculated in terms of maize production and fruits of columnar cacti**

Original vegetation type	% cover	Maize value	Fruit value	Total
Cardonal	0%	945.52	0.00	945.52
	2%	926.60	6.30	932.90
	10%	850.97	31.51	882.48
	25%	709.14	78.77	787.91
	47%	501.13	148.08	649.21
Jiotillal	0%	945.52	0.00	945.52
	2%	926.60	4.89	931.49
	10%	850.97	24.46	875.43
	25%	709.14	61.15	770.29
	47%	501.13	114.96	616.09
Tetechera	0%	945.52	0.00	945.52
	2%	926.60	2.28	928.88
	10%	850.97	11.38	862.35
	25%	709.14	28.45	737.59
	47%	501.13	53.49	554.62

## Discussion

Previous ethnobotanical studies in Quiotepec [24] found that gathering and consumption of wild plants continues playing an important role in local households’ subsistence. For instance, people consume nearly 74 edible wild plant species, which have a special role during seasons (or years) when agricultural products are scarce. Since the territory of Quiotepec is semiarid, columnar cacti represent some of the most abundant and culturally important resources from local forests, since these forests cover nearly 72.21% of the territory of Quiotepec.

The total potential annual production of fruits of the species studied is approximately 884.3 ton, with a total economic value of $947,852.75 U.S. dollars. From the total production, local people currently extract on average 0.17% of fruit (0.07% of *P. weberi,* 0.15% of *N. tetetzo,* 0.76% of *E. chiotilla,* and 0.11% of *M. geometrizans*), which suggests that the current extraction rates do not endanger the maintenance of populations and ecosystems where the populations occur. Examining the patterns of movement of people that harvest cacti products is relevant to evaluate the impact of fruit extraction. In the case studied, the areas submitted to extraction are relatively extent, especially because cacti produce few flowers and mature fruits per individual plant per day. This makes necessary for gatherers to visit a high number of individual plants to get a given amount of fruit per day. As discussed in a previous study [24], extraction of plant resources in general has less ecological impact when it occurs in an extended area than in focalized sites. Current use of products of columnar cacti represents nearly 3.11% of the total income obtained by a household from rainfed agriculture of maize, but this proportion could substantially increase under sustainable gathering systems.

Most people perceive that pollination is an interaction causing fruit production, but no specific pollinators are identified for *Pachycereus weberi* and *Neobuxbaumia tetetzo*. Bees have been observed visiting flowers of *Escontria chiotilla* and *Myrtillocactus geometrizans* and it is more explicit the perception of their role as pollinators. People also perceive that birds consuming cacti fruit are the “sowers of the mountains”, that is, they perceive the ecological importance of seed dispersal by birds. However, the general perception is that fruits from the mountain are abundant and no risk is associated to fruit collection. One of the main damage is associated to goats and cattle raising consuming or trampling seedlings and sapling plants, but this damage is not identified as a risk for cacti populations. Therefore, the communication of the information generated is a valuable issue which has been carried out directly by our research group to people and through local authorities of the Biosphere Reserve. From this information, the construction of local institution is taking place in order to planning routes and areas for goat raising.

Although perturbation analyses indicate that extracting 70% of fruits of *E. chiotilla* and 95% of those of *N. tetetzo* would allow λ values higher than 1, it is important to highlight that these harvest percentages cannot be considered sustainable. First, it should be considered that the projection matrices used for modeling population dynamics were based on information from populations different to those analyzed in this study. This is an attempt to make use of scientific information available for the area but we are conscious about its limitations. Although those populations are from neighboring areas, within the Tehuacán Valley, the environmental variations (mainly temperature and precipitation) associated to the study sites could be determinant for differences in seed germination, seedling recruitment, and plant survival and growth, which in turn influence the population dynamics. In addition, it is necessary to consider that the species studied are long-lived perennial plants, while data for projection matrices comprised one and two year’s records for *E. chiotilla* and *N. tetetzo*, respectively. This aspect is especially important since in the Tehuacán Valley as in other arid regions, inter-annual variations in temperature and precipitation are markedly high. Therefore, population dynamics models require data for more years. All these aspects limit the possibility to recommend the rates from the simulations performed. However, the demographic models constructed by previous studies demonstrated to be useful to perform simulations that constitute a reference point for constructing strategies, as discussed below.

Another important aspect to take into consideration is that other organisms belonging to the biotic community interact with the species studied and their survival depends on the resources provided by the columnar cacti. This is particularly the case of a number of species of frugivorous (birds, bats and other small mammals, and ants, among the most important groups), which can be affected by drastic reductions in the availability of fruits. In fact, the recruitment of new plants of columnar cacti depend on the specialized interaction with seed dispersers that consume fruits without destroying the seeds, but resting on shrubs and trees whose canopies are crucial for seedling establishment. In other words, a drastic reduction in the availability of fruits would determine a decrease in the probability that seeds reach sites secure for establishing.

There is scarce information on columnar cacti fruits removal rates by frugivorous, which would be of great importance to evaluate sustainability, from a biotic community perspective. Fleming and Sosa [42] documented that the rates of seeds removal from fruits of columnar cacti by *Leptonycteris* bats in the Sonoran Desert may be 10 to 80% per night, but diurnal dispersers and ants complete the activity. Godínez-Alvarez *et al.* [43] documented that bats remove nearly 75% of seeds from fruits of *N. tetetzo* in the Tehuacán Valley, whereas birds remove the remaining 25%. Munguía-Rosas *et al*. [44] documented that ants may deplete higher mass of *Pilososcereus leucocephalus* fruits than flying vertebrates. In general, studies on frugivory report that frugivorous consume practically all fruits of columnar cacti, although ethnobotanical studies in plantations of *Stenocereus stellatus* and in wild populations of *Polaskia chichipe*, report that part of the fruits are consumed by humans and domestic animals [22, 26]. In general, information about the amounts of fruits that are not removed remains uncertain.

Gathering rates from 50% to 10% affect relatively more and less, respectively, the community of fruigivorous feeding on columnar cacti fruits, whereas the economic benefit would be higher and lower, respectively. Therefore, an evaluation of such trade off from ecological and economic perspectives is necessary to identify a relatively more sustainable activity. As mentioned, the neat economic value of one hectare of maize is approximately $945.52 US dollars, and the ecological impact of the agricultural plot is the highest, removing in the more intensive system the entire plant cover, including younger stages of columnar cacti and their nurse plants, necessary for a slow recovery of vegetation. On the other hand, gathering all fruits per hectare would determine lower economic benefits ($315.00 U.S. dollars for cardonal, $244.60 for jiotillal, and $113.80 for tetechera) than the agricultural system, and a high ecological impact on the community of frugivorous.

Intermediate gathering rates in non-cleared forests would determine lower economic benefits, but also lower ecological impact, in a proportion depending on the amount of fruits extracted. Considering all ecological and socio-economic indicators, the forest systems with gathering harvesting rates of 25% of fruits show the relatively higher levels of sustainability (Figures 4, 5, and 6). However, the economic benefits are markedly lower than those determined by rainfed agriculture, and therefore, this system have low incentives to be practiced instead of agriculture. Systems involving gathering rates of 50% of fruits are relatively less sustainable than those extracting 25% fruits and do not have the possibility to resemble the economic benefit of agriculture. Possible alternatives can be developed maintaining low gathering rates in more extended areas (for instance, extraction rates of 25% of fruits in more than one hectare), but determining the technical viability of these systems require evaluations in field in relation to time frame and effort needed to put them in practice. Such alternatives could be important, considering that clearing land for agriculture cause tensions between ejidatarios and comuneros; therefore, systems of forest harvest could have higher social benefit than agriculture alone.

Intermediate impact through agroforestry systems maintaining part of the vegetation cover would determine lower production of maize per hectare, but could be compensated by the economic value of non-timber forest products. According to [38–40], agroforestry systems in the Tehuacán Valley vary highly in their capacity to maintain vegetation cover, from 2 to 47% of their area. In principle, it is possible to expect that the reduction of the economic benefit from maize and an increment of columnar cacti fruit could be proportional to the amount of vegetation cover maintained. As more as vegetation cover is non-cleared, the lower the amounts of maize and the higher the amounts of fruit are expected. According to Table 11, the economic value of the agroforestry system products is lower than that estimated for the agricultural system without any vegetation cover. However, our analysis considered only four species of the forest system, but in a previous study [24] we identified 38 useful plant species for cardonal, 44 for jiotillal, and 46 for tetechera forests. Further analyses of the economic value of all those resources would help to a more precise estimation of the economic value of agroforestry systems.

The different scenarios modeled should be considered for the time being, only as feasible bases for designing sustainable gathering strategies of the plant resources analyzed, but their construction require deeper ecological and economic research, pilot experimental gathering parcels, and technical innovation guided by adaptive management approaches [45, 46]. For the moment, the scenarios modeled in this study are only proposals that aspire to be bases for workshops with people of Quiotepec and authorities of the Biosphere Reserve, in order to design appropriate viable management techniques. Adaptive management can be a methodological approach useful to guide actions with the information available, implementing a rigorous monitoring system of the result of the interventions designed, and developing efficient strategies to learn from the experiences conducted. Such approach is similar to the philosophical bases of “framework for sustainability evaluation of natural resources management systems” [37], which visualize sustainability as a process of construction based on analyzing the systems intervened after each step of intervention. One virtue of MESMIS is also identifying strong and weak aspects of a system, in order to define specific strategies for solving particular problems of weakness [37]. For instance, in relation to the economic benefits the systems under different gathering scenarios could be strengthened through activities directed to transform fruits into manufactured products. In the region there are available techniques to dry fruits (“pasado” or raisins of fruits), to prepare jellies, liquors and wine with pulp, or a butter-like paste with grounded seeds, or cooked flower buds preserved in vinegar. All these local techniques could be the bases for strategies to increase economic benefits of the systems.

Plant and ecosystems management are expressions of knowledge, practices, and believes that form part of a human culture. In the specific cases studied, symbolic factors associated to cacti fruit were not analysed and would be an interesting research topic for a deeper understanding of the management system. Gathering of cacti fruits is a very ancient activity practiced in the region still coexisting with agriculture and other economic activities. Cacti fruits and other forest resources are directly consumed and some of them are sources of monetary incomes. These resources represent a complementary option but may become more important as long as the markets increase demand of particular products. This has been the case of fuel wood, agaves for mescal production and other products. NTFP are collective resources whose use may require regulations for a sustainable ways of use.

Gathering of non-timber forest products like those analyzed in this study may have a lower impact compared with other economic activities, such as agriculture, livestock and illegal extraction of ornamental plants occurring in the area. Designing integral efficient agro-silvo-pastoralist systems based on the experience of local people could be an appropriate alternative and an important challenge for sustainability in rural areas of the arid Tehuacán Valley. These strategies can be technically viable and ecological information may be a strong support for designing such systems. However, in addition to techniques ecologically friendly, efforts to construct and developing fair trade markets are crucial to support actions for long-term maintenance of ecosystems. The technical viability is one part of the problem to solve, but at the end, secure and fair routes of commercialization of forest products are needed to construct sustainable scenarios of non-timber forest products use. Working with local people, ethnobiologists and ecologists may contribute to construct technically sustainable management models, but economists and local and regional authorities should play a more substantial role in supporting fair the improvement of the quality of life of rural people living in the Tehuacán Valley.

## Authors’ information

EPN postgraduate student and Academic Technician at the Centro de Investigaciones en Ecosistemas (CIEco), UNAM. PD full time researcher at the UBIPRO, Facultad de Estudios Superiores (Faculty of Higher Studies), UNAM. AC full time researchers at CIEco, UNAM.
